# Influence of Thin Film Deposition on AFM Cantilever Tips in Adhesion and Young’s Modulus of MEMS Surfaces

**DOI:** 10.3390/ma15062102

**Published:** 2022-03-12

**Authors:** Pedram Heidari, Majid Salehi, Behrooz Ruhani, Violeta Purcar, Simona Căprărescu

**Affiliations:** 1 Department of Mechanical Engineering, Najafabad Branch, Islamic Azad University, Najafabad 8514143131, Iran; pedram.heidari90@gmail.com (P.H.); Salehi.majid89@gmail.com (M.S.); b.ruhani55@gmail.com (B.R.); 2 National Institute for Research & Development in Chemistry and Petrochemistry—ICECHIM, Splaiul Independentei No. 202, 6th District, 060021 Bucharest, Romania; 3 Faculty of Applied Chemistry and Materials Science, Department of Inorganic Chemistry, Physical Chemistry and Electrochemistry, University Politehnica of Bucharest, Ghe. Polizu Street, No. 1-7, 011061 Bucharest, Romania; simona.caprarescu@upb.ro

**Keywords:** AFM tip deposition, adhesion force, Young’s modulus, thin film, microelectromechanical systems, coating

## Abstract

Adhesion is a critical factor in microelectromechanical systems (MEMSs) and is influenced by many parameters. In important fields, such as microassembly, an improved understanding of adhesion can result in higher precision. This study examines the influence of deposition of gold and titanium onto the atomic force microscope (AFM) tips in adhesion forces and Young’s modulus, between a few MEMS substrates (silicon, gold, and silver) and the AFM tips. It was found that, except for gold substrate, an AFM tip coated with gold has the highest adhesion force of 42.67 nN for silicon substrates, whereas the titanium-coated AFM tip decreases the force for all the samples. This study suggests that such changes must be taken into account while studying the adhesion force. The final results indicate that utilizing gold substrate with titanium AFM tip led to the lowest adhesion force, which could be useful in adhesion force measurement during microassembly.

## 1. Introduction

Surface forces are among the most important challenges in miniature structures’ design and function. Tribological aspects such as stiction, adhesion, friction, and wear receive significant attention in developing and manufacturing micro- and nanostructures, such as microelectromechanical systems (MEMSs). These aspects are becoming increasingly important for structures with a large surface-area-to-volume ratio [1,2,3]. For instance, surface micromachining has been widely employed to manufacture microstructures. During the release process, if the restoring force is less than the attractive force, the structure tends to adhere to the underlying substrate. This phenomenon is commonly referred to as stiction [4,5]. Since microstructures placed in vertical positions can adhere to the substrate and disturb the entire structure [6], many studies have been conducted to dominate the surface forces at micro- and nanoscale in recent years. Shui et al. [7] proposed a method to continuously regulate adhesion forces by creating mechanical microvibrations for a typical polydimethylsiloxane (PDMS) glass substrate. Their result indicated that the adhesion force could be increased or weakened 77 times using this technique. In another study, Peillon et al. [8] investigated the changes in surface forces by depositing tungsten microparticles on tungsten substrates with different degrees of roughness. Theoretical and experimental methods were utilized to measure the forces, and they found that the dependence of adhesion force on particle size has a secondary effect, compared with surface roughness. Moreover, the assembly of these microdevices is greatly dependent on the existence of adhesion forces because they can create a considerable challenge in manipulating micro-objects [9]. For example, Kim et al. [10] designed an automated two-fingered microhand to solve the problem of adhesion forces in micromanipulation. To this end, a microhand having dexterous motion was used to grab an object. Local stream and dynamic releasing methods were utilized to detach the object adhered to one of the end effectors. The mechanisms of adherence confirmed that Van der Waals forces are responsible for surface tension. This obstacle was scrutinized, and as a solution, rough and hydrophilic surfaces were proposed [11,12,13]. The deposition effect and the resulting changes on the surface that can be effective in the adhesion force analysis have been assessed in many studies [14,15,16,17]. Sabooni et al. [18] analyzed the adhesion strength of steel substrates through the deposition of ZnMg–Zn bilayer coatings. They used the thermal evaporation method for the deposition process. Their results suggested that the thicknesses of the coated layers, as well as the interfacial adhesion strength, are important parameters in adhesion performance. Zhang et al. [19] used CrN as a thin film and various substrates to understand the adhesion mechanism after CrN deposition. Scratch and indentation tests were applied, and it was found that the substrate’s modulus is a key factor in adhesion analysis. In another research, Isaifan et al. [20] examined the impact of surface roughness after deposition of antidust titanium thin films on the capillary, Van der Waals, electrostatic, and gravitational forces. Their results indicated that surface roughness and antidust coatings could reduce the adhesion force.

The atomic force microscope (AFM) is commonly used for the calculation of adhesion force. It is a high-powered tool that provides researchers with high-resolution measurements, manipulating individual atoms, micro- and nanoassembly, and 3D imaging of the surface [21]. Using the AFM is superior to other technics of adhesion force measurements, as it is capable of acquiring forces with high sensitivity and can cover a wide range of topics [22,23]. This microscope is equipped with a cantilever and a tip. When the cantilever is scanning the surface, it is deflected due to interaction forces between the sample and the AFM tip. This deflection and the distance between the cantilever and the sample can be measured using a laser and a detector; a force–distance curve can also be derived to measure adhesion forces [24,25,26]. Kim et al. [27] suggested a design methodology for the control of adhesion forces for nanostructures with the help of force–distance curve measurements using AFM. They engineered several nanostructures by laser interference lithography, followed by deep reactive ion etching, and compared the results with bare surfaces. The characterized adhesion force using the force–distance curve showed that the engineered nanostructures’ adhesion force was smaller than that of the bare surface. Individual aerosol particles’ adhesion force was studied by Ono et al. [28] through the force–distance curve. Considering the distribution of adhesion force, the collected data from multiple points on a single particle reported that a force–distance curve can be applied for the direct measurement of adhesion force.

However, the influence of AFM tip and silicon substrate modification on measuring adhesion forces in MEMS surfaces as a way of controlling or understanding the adhesion mechanism is not yet fully understood. As a result, in this paper, we sought to investigate the mutual impact of both silicon substrate and tip thin film deposition on adhesion forces. To this end, Au and Ti thin films were coated on the AFM tips, and then Ag and Au were deposited on silicon substrates. The data and methods of the deposition process are explained in the second section. The adhesion force measurements were considered for this study concerning the minimum force needed for the AFM tip to be separated from the sample. The experimental investigations, conducted using a force–distance curve, focused on determining the adhesion force between the different tip and substrate materials commonly used in MEMS. In the third section, two types of investigation are presented. The first type was conducted to indicate the effect of variation in the AFM tip and substrate materials on adhesion forces. The second type of research was carried out to determine the effect of AFM tip deposition on Young’s modulus and evaluate its effect on adhesion forces. In the last section, the conclusions of the study are presented.

## 2. Materials and Methods

**Figure 1:**
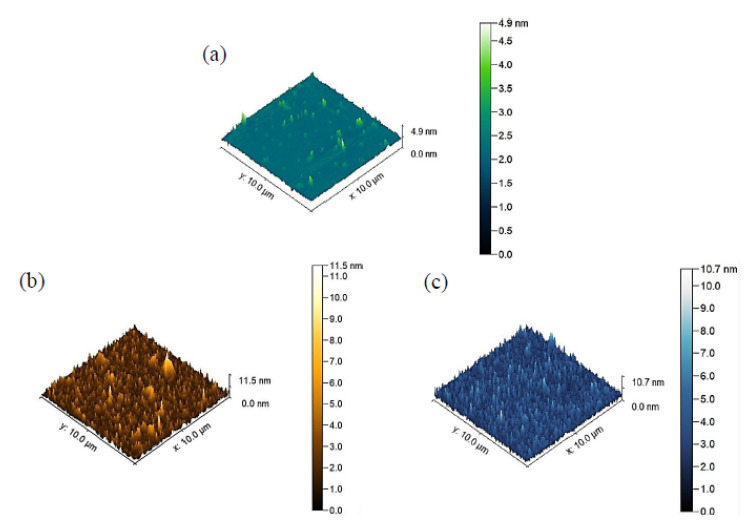
Three-dimensional topographies of the (**a**) uncoated silicon surface, (**b**) gold surface, and (**c**) silver surface from Gwyddion software (version 2.59).

In this paper, the analyzed materials are thin films of silver and gold, with a thickness of 500 nm. The thin films consisted of one layer of each material deposited on silicon Si <100>. One uncoated silicon substrate was used as well. The 3D and 2D silver surface of approximately 10 µm × 10 µm is given in Figure 1. Thin films of gold and titanium with a thickness of 30 nm were deposited by the magnetron sputtering method on AFM tips. Additionally, to compare the results and understand the effect of tip deposition on adhesion force, one regular and common uncoated silicon AFM tip without any deposition was used. These materials were selected due to their common use in MEMS structures [29]. A grid of 16 squares was considered for each sample, and the adhesion force was determined for the center of each square. In each square, a sufficient number of force–distance curves was taken using three different AFM tips.

### 2.1. Coating of Substrates

The materials employed for the deposition were composed with the highest purity: gold 99.99% and silver 92.5%. Prior to any deposition, each silicon substrate was rinsed. First, the substrates were washed with water and soap. Then, acetone, isopropanol, dichloromethane, ethanol, methanol, acetone, and deionized water were used to rinse the substrates. Finally, to dry the substrates, pressured nitrogen gas pressure was utilized instantly. The deposition of silver and gold on silicon substrates with thicknesses of 500 nm was accomplished by the thermal evaporation method. To this end, first, rotary and turbopumps were employed to evacuate the chamber and maintain the pressure below 6 × 10^−6^ millibars. A quartz crystal microbalance was used to measure the coating thickness. In order to keep the substrate temperature consistent, the 40 °C cold-water circulations were utilized. The distance between the evaporator and the substrate was approximately 13 cm, and it was kept fixed. Various evaporation bushes, such as molybdenum, tungsten, tantalum, and graphite, could be selected depending on the type of evaporation material. The quartz crystal device was observed to have a nominal thickness measurement accuracy of ±1 Å and an evaporation rate with an accuracy of 0.1 Å. It should be mentioned that the deposition rate was kept at 0.1 nm/s during the coating.

### 2.2. Experimental Procedure

The features of the thin films of substrates were accomplished using AFM Nano wizard II (JPK Company, Berlin, Germany) and FESEM MIRA 3 (TESCAN Company, Brno-Kohoutovice, Czech Republic). Both types of research were conducted at a relative humidity of 45% and a temperature of 22 °C. The cantilevers used for these tests were HYDRA cantilevers (AppNano Company, Mountain View, CA, USA), and as stated by the manufacturer’s specifications, had the length of 100 µm, a width of 18 µm, a thickness of 0.6 µm, a spring constant of 0.292 N/m, and a resonance frequency of 66 kHz. The tip of these cantilevers had a radius smaller than 10 nm.

**Figure 2:**
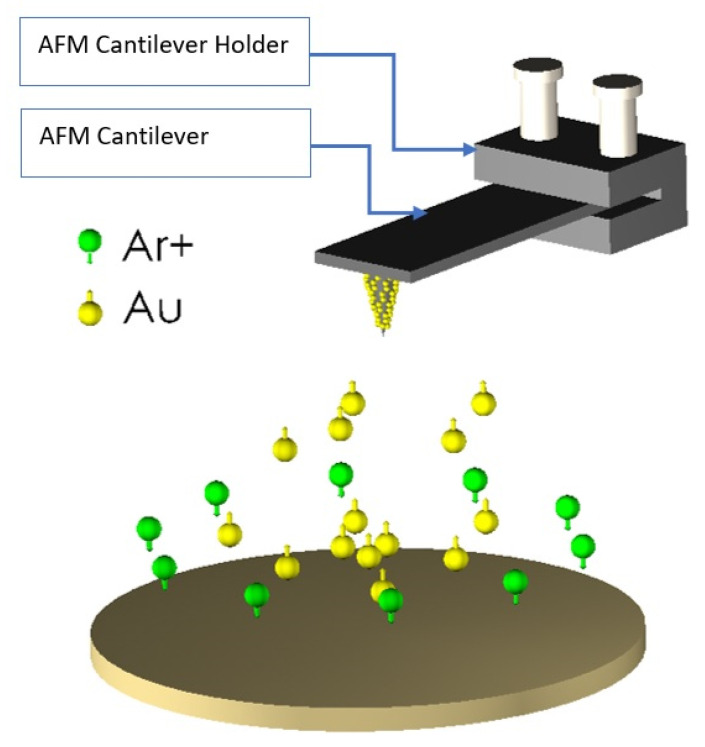
Schematic image of the magnetron sputtering method to deposit Au on AFM cantilever tip.

Gold and titanium with a thickness of 30 nm were deposited on cantilever tips to determine any change in adhesion forces and Young’s modulus between the AFM tip and substrate materials. The deposition of AFM tips was achieved by the magnetron sputtering method. A quartz crystal was operated to accurately control and measure thin films’ thickness. The data of this deposition are summarized in Table 1. Concerning this table, when the chamber is vacuumed, the neutral argon gas enters the chamber, resulting in chamber electrical discharge, argon ionization, and positive ions creation. While the energy and momentum are assigned to the atoms, the ions collide with the surface target. The violent collision of energized ions with the target emits target metal atoms into space. Eventually, these metal atoms are deposited on the cantilever tip, creating a metallic film. The target is cooled by water, which results in smallish radiation heat generation [30]. Additionally, Figure 2 schematically shows an AFM cantilever tip coated with gold via the magnetron sputtering method.

**Figure 3:**
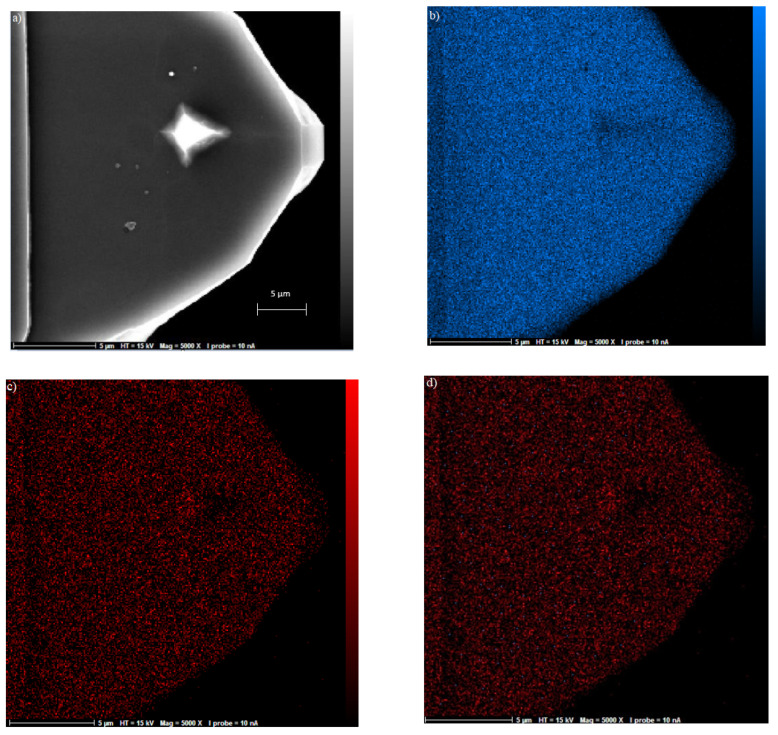
(**a**) SEM image of the cantilever tip with the scale of 5 µm; (**b**) EDX image showing the dispersion of Si on cantilever; (**c**) EDX image showing the dispersion of 90.18% gold and (**d**) dispersion of both gold and Si on the cantilever tip.

The cantilever tip images were obtained using scanning electron microscopy (SEM, Delmic BV, Delft, Netherlands) and energy-dispersive X-ray spectroscopy (EDX, Hitachi, Virginia, USA) in order to confirm the deposition process. For instance, Figure 3a shows an SEM image of the cantilever tip, and Figure 3b shows an EDX image of the silicon tip without any deposition. Later, a layer of gold with 90.18 wt% was deposited on the AFM tip (Figure 3c); Figure 3d shows an AFM tip with both gold and silicon.

**Figure 4:**
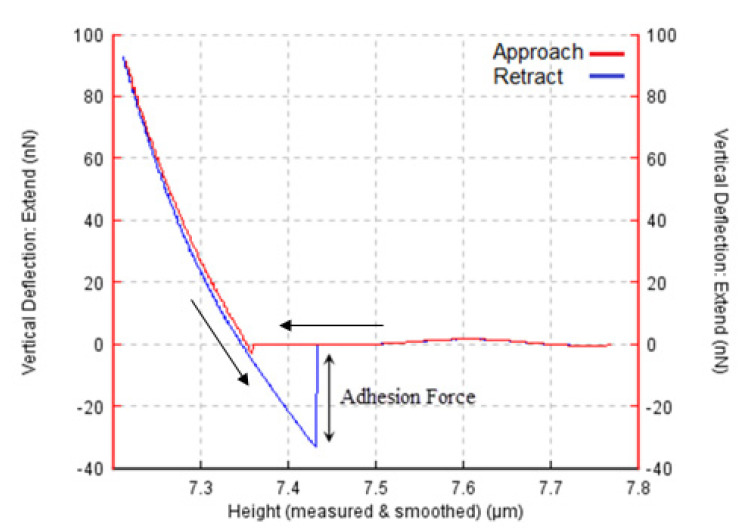
The force-distance curve of the silver substrate with gold-coated AFM tip.

A force–distance curve was used to measure adhesion forces, and AFM was utilized in contact mode. Figure 4 is a representative of one force–distance curve of the silver substrate with a gold AFM tip. In this curve, first, the cantilever approached the surface, but the AFM tip did not have any contact with the surface. Next, the tip jumped toward the surface because of the existing attractive forces. Then, the tip wanted to leave the surface, but there existed adhesive forces, so the tip was required to overcome these forces and snap off from the surface (here, the adhesion force is attained). Eventually, the cantilever returned to its starting position. In order to calculate and analyze adhesion forces better, one substrate was chosen, and then AFM tips with different materials were applied on the surface. For each substrate, three different areas were selected, and in each area, force–distance curves were obtained, and finally, the average adhesion force was derived. The same method was used to conduct the second type of investigation, which studied the effect of tip deposition on Young’s modulus and the effect of this change on adhesion force.

## 3. Results and Discussion

### 3.1. Adhesion Force

The first investigation was conducted to determine the effect of variation in the AFM tip and substrate materials on adhesion forces. Nine measurements were performed, and the calculation of adhesive force was carried out at several points to improve the reliability of the measurements.

Gold, silver, and silicon surfaces make close contact with AFM tips from titanium, gold, and silicon. Uncoated silicon AFM tips are the most common ones used in different types of research, and in this investigation, comparisons were made with silicon tips to analyze the results. Except for the gold surface, when other surfaces made contact with the gold AFM tip, adhesion force increased, compared with the uncoated silicon tip. When the gold tip scanned the gold surface, the adhesion force decreased from 10.12 to 7.48 nN, but in silver and silicon surfaces, the values increased from 11.17 to 27.78 nN, and from 25.67 to 42.67 nN, respectively (Table 2). The reason for this exception might be related to the surface roughness of Au substrate, which is the highest among the others (11.50 nm versus 10.70 nm for Ag and 4.9 nm for Si). When this rough surface was scanned with an uncoated Si probe, it showed different behavior in comparison with again rough Au probe. On the other hand, the titanium AFM tip decreased the adhesion force in all samples. It is worth mentioning that the shape of the tip at the apex is influential in adhesion force measurement. If the tip radius is smaller, then the tip–sample contact area is smaller, and therefore, the measured adhesion is lower. It seems that the AFM tip coated with titanium obtained a less radius at the apex after the deposition process than the Au probe, and this is why the titanium tip was measured as having lower adhesion. Additionally, the reason for the noticeable difference in adhesion results between Au tip on Si substrate (42.67 nN) and Si tip on Au substrate (10.12 nN) can be the greater radius of the Au tip than that of the Si tip.

Surface energy that changes with the deposition process (considering as a fundamental parameter in measuring the adhesion forces) can be regarded as an interpretation of the adhesion forces’ results. The inverse relationship between surface energy and adhesion has been reported in previous studies [2,16]. Since the present research focused on modifying the AFM tip and substrates by coating thin films, surface energy was analyzed using silicon as the based material. All of the films were coated on silicon, in both substrate and tips. For investigating the effect of surface energy first, this parameter was calculated for tip materials, which were Si–Si (uncoated), Si–Au (30 nm of Au on Si tip), and Si–Ti (30 nm of Ti on Si tip). Then, the surface energy of substrates, Si–Au (500 nm of Au on Si), Si–Ag (500 nm of Ag on Si), and Si–Si (un-coated), were measured. The tip and substrate surface energy values are given in Table 3. By comparing these values with the measured adhesion force, it can be concluded that these two parameters had a reverse relationship. For instance, when Au tip with Si–Au structure was in contact with Au substrate with Si–Au structure and 2.7 J/m^2^ surface energy, the adhesion was 10.12 nN. When this probe scanned Ag substrate with Si–Ag structure, the surface energy lessened to 1.5 J/m^2^, but the adhesion increased to 11.17 nN. The same trend can be observed in other tips and substrates.

Another factor that plays an essential role in interpreting adhesion force measurements besides surface energy is the Hamaker constant. Van der Waals force is actually an interaction between atoms or molecules. One approach to calculate Van der Waals force is Hamaker. The Hamaker constant is a coefficient considering the Van der Waals interaction between two materials, and it has a firm association with adhesion. The Hamaker constant relies on the microscopic feature of two interacting atoms and the strength of the interaction between bodies and the medium covering them [34]. The Hamaker constant of two different materials can be calculated as follows [16]:

(1) $$A_{H_{12}}=\sqrt{A_{H_{11}}A_{H_{22}}}$$

where $A_{H_{11}}$ represents the Hamaker constant of substrate, and $A_{H_{22}}$ denotes the Hamaker constant of tip material. Table 4 reports the calculated values of Hamaker coefficients of the above substrate and tip materials.

By comparing the values between Hamaker coefficients and adhesion forces, we can conclude that except for silicon substrate, whenever the Hamaker coefficient between each sample increased, the adhesion force increased as well. On the other hand, decreased Hamaker coefficient caused diminished adhesion force. The exception of silicon is because no materials were coated on the Si substrate, and this surface was smoother than 500 nm Au- and Ag-coated substrates. When the tip and substrate come in contact, the roughness of the substrate has a more substantial share in determining the Hamaker coefficient due to its larger size. The Hamaker coefficients of the materials obtained from the present study and the relationship of it with adhesion force are generally consistent with previously published results [35,36].

### 3.2. Young’s Modulus

The second type of research was accomplished to study the influence of tip deposition on Young’s modulus and subsequently on adhesion force. Young’s modulus is one of the parameters that must be taken into account for measuring adhesion forces. This parameter is related to two bodies when they come close to each other, so when the AFM tip and surface sample come into contact, Young’s modulus can directly affect the adhesion force. This parameter is used in mathematical models for the calculation of adhesion forces [20]. It is also considered from the experimental point of view, and it is possible to obtain it through the use of nanoindentation [37] or AFM. For example, in [38], the AFM was used to measure Young’s modulus in soft and thin samples such as polymers. Any change in the surface can affect Young’s modulus. Therefore, when the deposition occurs on the AFM tip, this change must be considered in measuring adhesion forces [39]. In this experiment, the AFM tip was directly utilized to obtain Young’s modulus. Table 5 presents the values of Young’s modulus based on the different tip and substrate materials. According to the obtained results, it can be found that by changing the tip materials, the values of Young’s modulus also changed since the values of silicon tip are quite different from gold and titanium tips.

**Figure 5:**
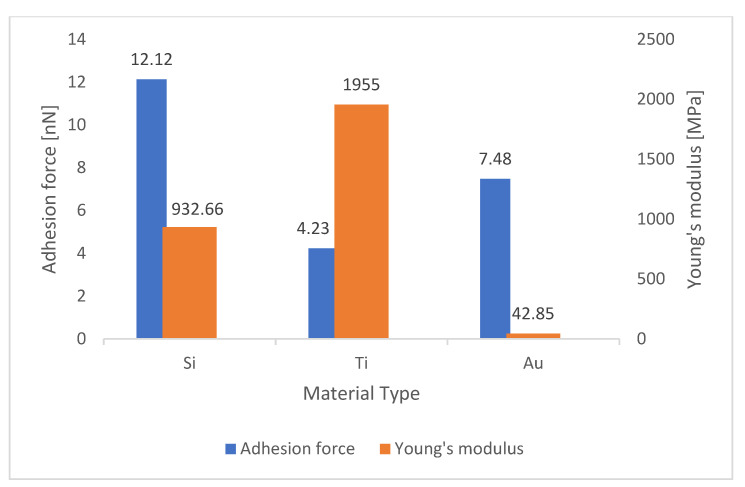
Comparison of values of Young’s modulus with adhesion force in the gold surface.

**Figure 6:**
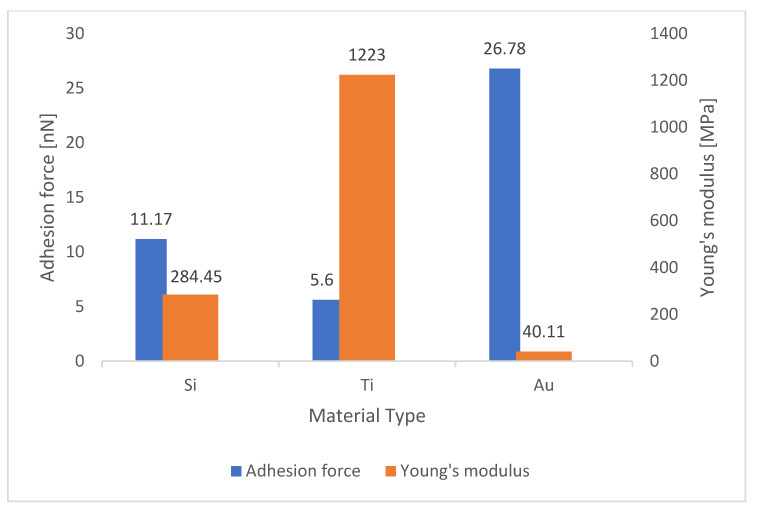
Comparison of values of Young’s modulus with adhesion force in the silver surface.

**Figure 7:**
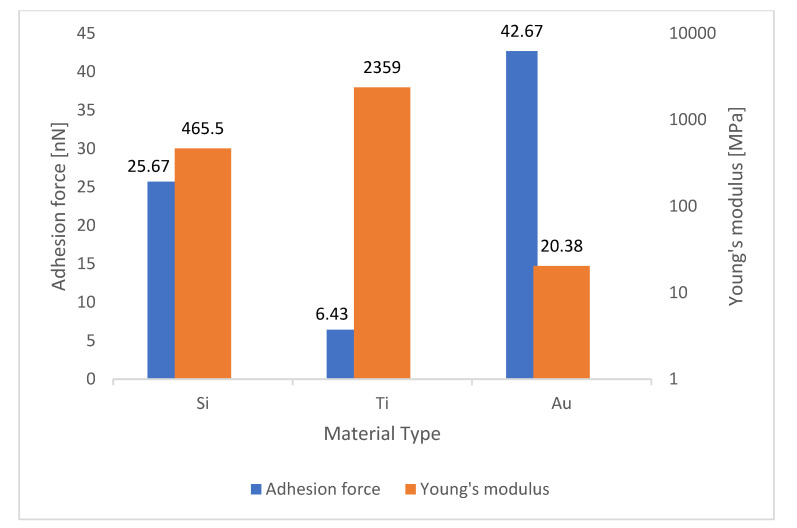
Comparison of values of Young’s modulus with adhesion force in the silicon surface.

To investigate any relationship between this parameter and adhesion force, these values were compared with those obtained in Table 2, and then column charts were drawn for each substrate. As it can be seen in Figure 5 in the gold surface, by using silicon and titanium tips, when Young’s modulus increased from 932.66 to 1955 MPa, the adhesion force decreased from 10.12 to 4.23 nN, and when the gold tip scanned the surface, Young’s modulus decreased from 1955 to 42.85 MPa, while the adhesion force increased from 4.23 to 7.48 nN. Figure 6 shows that in the silver surface, by using silicon and titanium tips, Young’s modulus increased from 284.45 to 1223 MPa, but the adhesion force decreased from 11.17 to 5.60 nN. On the other hand, when a gold AFM tip came in contact with a silver surface, Young’s modulus decreased from 1223 to 40.11 MPa, while the adhesion force increased from 5.60 to 26.78 nN. Additionally, the exact inverse relation for silicon surface can be seen in Figure 7.

Considering Table 5 and Figure 5, Figure 6 and Figure 7, it can be concluded that increasing Young’s modulus reduced the elasticity of the cantilever, resulting in a very small beam deflection in the AFM tip and a much lower adhesion force calculated by the cantilever.

## 4. Conclusions

This study investigated the influence of specific depositions on an AFM tip on adhesion forces and Young’s modulus to improve the control of adhesion force in MEMS application, especially in the microassembly. Titanium and gold were selected as thin films for cantilever tips, and silver and gold were deposited on silicon substrates. The results are summarized as follows:

- The deposition on the AFM tip was found to influence the adhesion force; however, the influence varied with the material of deposition on the tip and the substrate;
- The adhesion force was strongly dependent on the surface energy of both substrate and the AFM tip;
- The Hamaker constant between substrate and AFM tip was critical in adhesion force measurements. Considering that it is constant, a direct relation between the Hamaker coefficient and adhesion force in different surfaces can be established;
- The use of an AFM tip coated with gold increased the adhesion force except for gold substrate. However, using titanium-coated AFM tips could decrease the adhesion force in the case of all surfaces;
- Young’s modulus bore an inverse relationship with adhesion force for various combinations of tip and substrate materials. Increasing Young’s modulus reduced the elasticity of the cantilever, resulting in a very small beam deflection in the AFM tip and a much lower adhesion force calculated by the cantilever;
- Deposition on the substrate’s surface or AFM tip can affect the surface energy, the surface roughness, and Young’s modulus, which are the main parameters for the characterization of adhesion force.

## Figures and Tables

**Table 1:** Data of materials deposition by the magnetron sputtering method on the AFM tips.

Deposited Material	Argon Gas Pressure (mbar)	Final Pressure (mbar)	Voltage (V)	Temperature (°C)	Deposition Rate (Å/s)	Target-Substrate Distance (mm)
Gold	6.4 × 10^−3^	2.5 × 10^−5^	500	54	2.43	130
Titanium	6.4 × 10^−3^	2.5 × 10^−5^	750	54	1.7	130

**Table 2:** Adhesion forces’ values of substrates obtained by various tip materials.

Substrate Material	Adhesion Force Based on the Tip Material (nN)		
	Gold	Titanium	Silicon (Uncoated)
Gold	7.48	4.23	10.12
Silver	26.78	5.60	11.17
Silicon	42.67	6.43	25.67

**Table 3:** The tip and substrates’ values of surface energy [31,32,33].

Tip Materials	Surface Energy (J/m^2^)	Substrate Materials	Surface Energy (J/m^2^)
Si-Si	0.26	Si-Au	2.7
Si-Au	2.7	Si-Ag	1.5
Si-Ti	1.16	Si-Si	0.26

**Table 4:** Hamaker coefficients of surfaces.

Surface	Hamaker Coefficient (*A*/10^−20^ J) $A_{H_{12}}$
Au–Au	40.00
Au–Ti	32.86
Au–Si	35.55
Ag–Au	44.72
Ag–Ti	36.74
Ag–Si	39.75
Si–Au	35.55
Si–Ti	29.12
Si–Si	31.60

**Table 5:** Values of Young’s modulus of substrates obtained by various tip materials.

Substrate Material	Young’s Modulus Based on the Tip Material (MPa)		
	Gold	Titanium	Silicon (Uncoated)
Gold	42.85	1955	932.66
Silver	40.11	1223	284.45
Silicon	20.38	2359	465.50

## Data Availability

The data are not publicly available due to their containing information that could compromise the privacy of research participants.
